# Retinal arterial–venous pulse delay: a new specific marker for a carotid–cavernous fistula

**DOI:** 10.3389/fopht.2023.1301410

**Published:** 2023-12-11

**Authors:** Edward F. Linton, Thomas R. Tedeschi, Noor-Us-Sabah Ahmad, Jui-Kai Wang, Randy H. Kardon

**Affiliations:** ^1^ Department of Ophthalmology and Visual Science, University of Iowa, Iowa City, IA, United States; ^2^ Rehabilitation Research and Development, Iowa City Veterans Affairs (VA) Health Care System and Veterans Affairs (VA) Center for the Prevention and Treatment of Visual Loss, Iowa City, IA, United States; ^3^ Regeneron Genetics Medicine – Ophthalmology, Regeneron Pharmaceuticals Inc., Tarrytown, NY, United States

**Keywords:** laser speckle flowgraphy, ocular blood flow, carotid cavernous fistula, non-invasive diagnostics, retinal arterial-venous pulse delay

## Abstract

**Purpose:**

The purpose of the study was to describe ocular blood flow changes in eyes affected by a carotid–cavernous fistula (CCF) using laser speckle flowgraphy. We hypothesized that imaging blood flow velocity waveforms in the retinal arterioles and venules simultaneously would reveal specific characteristics of an arteriovenous (AV) connection.

**Design:**

The study was an observational case series, with a retrospective case–control analysis.

**Methods:**

Five patients with a CCF underwent measurement of ocular blood flow using laser speckle flowgraphy. The blood flow was compared retrospectively between a control group of healthy subjects (*n* = 32) and patients with an elevated intraocular pressure or venous outflow impairment without an AV fistula (*n* = 40). The outcomes were derived from the arteriole and venule blood flow velocity waveforms, including an A–V phase delay and flow pulsatility.

**Results:**

The presence of an active CCF was associated with an increased delay in the peak velocity measured in the retinal venule (10.7% ± 2.2% of the cardiac cycle duration) compared with unaffected fellow eyes (1.8% ± 0.2%; *p* = 0.05) or control eyes of normal subjects (2.7% ± 0.3%; *p* = 0.02). This delay disappeared after fistula thrombosis and was not present in eyes with a central retinal vein occlusion (CRVO), glaucoma, non-arteritic anterior ischemic optic neuropathy (NAION), or papilledema. The venule blood flow velocity decreased during systole (and in some cases momentarily stopped), leading to a delayed pulse with a greater amplitude in the venules than in fellow eyes and normal controls after normalizing to the arteriole amplitude (1.71 ± 0.3 vs 0.54 ± 0.03 vs 0.59 ± 0.02; *p* = 8.0E-12). This specific AV delay could also be identified in a scanning laser ophthalmoscope (SLO; SPECTRALIS®) video.

**Conclusion:**

Laser speckle flowgraphy reveals dynamic retinal vascular changes in eyes affected by a CCF, which are not present in healthy controls or patients with other eye conditions, and which reverses with treatment.

## Introduction

Indirect carotid–cavernous fistulas (CCFs) are abnormal connections between the carotid arterial system and the cavernous sinus through one or more small meningeal branch vessels. Arteriovenous (AV) shunting causes a wide variety of symptoms (1–5), through venous congestion and reduced AV pressure gradients. Vision loss is the major morbidity, predominantly through elevated intraocular pressure (IOP) or ischemic complications (4–6). The gold standard for CCF diagnosis is digital subtraction angiography, which is invasive and costly (4–7). Venous blood from the retina typically drains into the cavernous sinus via the superior ophthalmic vein. This valve-less connection exposes ocular structures to hazards from a CCF, and also creates a diagnostic opportunity (7, 8). AV fistulas elsewhere introduce changes in pressure and velocity waveforms that can be measured in veins upstream of the connection (9). Non-invasive imaging of ocular blood flow can be performed through several methods (10, 11), with trade-offs between spatial and temporal resolution.

Laser speckle flowgraphy (LSFG) is an imaging technique that enables simultaneous measurement of a relative blood flow velocity pulse waveform in the optic nerve, retina, and choroid using the laser speckle phenomenon. A pseudorandom speckle pattern is emitted by a diode laser, and red blood cell movement blurs the pattern reflected back to the sensor (12). A single previous report of LSFG in CCF showed reduced choroid flow (13), but the technology’s ability to record from the arterioles and venules simultaneously with high temporal resolution has the potential to reveal unique flow patterns indicative of an AV shunt.

Given the diagnostic challenge that a CCF can present (1–3, 5, 14, 15), the invasiveness of angiography and the propensity for spontaneous resolution (4, 16, 17), rapid non-invasive diagnostics are desirable for diagnosis and for differentiating an active fistula from one that is thrombosing. Goldmann applanation tonometry of the affected eye often reveals a significant change of IOP between systole and diastole during the cardiac cycle. Orbital Doppler (18, 19), enhanced depth imaging ocular coherence tomography (13), indocyanine green angiography (10), and, historically, pneumoplethysmography (20, 21) have all demonstrated changes related to increased pulsatility, venous hypertension, or turbulent flow in individual reports and small case series. Even so, many cases go months or years without a diagnosis, and diagnostic uncertainty can lead to unnecessary invasive imaging.

The studies investigating the use of blood flow measurements with LSFG in different clinical states have been rapidly increasing in recent years (6, 12, 22). Investigators have applied LSFG to assess optic nerve head circulatory function in normal subjects (23, 24), glaucoma (25, 26), normal-tension glaucoma, and non-arteritic ischemic optic neuropathy (NAION) (27). The mean blur rate (MBR) is the principal measurement in LSFG; it is a quantitative index of blood flow velocity measured simultaneously in the retina, choroid, and optic nerve head, which can then be calculated for any region of interest in the 4-second video acquired during four heart beats. We hypothesized that the pulsatile waveform of the relative flow volume (RFV), an MBR-derived measurement of volume blood flow (28), would provide new ocular hemodynamic information between the eyes and individuals (29), which would in turn reveal characteristics specific to CCF.

## Materials and methods

We obtained blood flow measurements from consecutive cases of clinically diagnosed indirect CCF. We were open to inclusion of direct CCF cases, but none presented in the timeframe stable enough to come to the clinic for enrollment. The LSFG images were acquired at presentation in all cases and following resolution when possible. The demographic information, as well as details of each patient’s presentation, course, and outcomes were recorded.

Our group has been studying ocular blood flow in a number of eye conditions over the last decade, so we were able to compare these consecutive CCF cases with existing data from healthy controls and in other conditions with some pathophysiologic overlap with CCF. We retrospectively reviewed LSFG ocular blood flow measurements from healthy control subjects and patients affected by acute NAION, papilledema, central retinal vein occlusion, and glaucoma. The demographic information for each group of subjects was collected. The participants had undergone imaging in both eyes during at least one study visit. The testing protocols and subject selection were carried out in accordance with the Declaration of Helsinki. All subjects gave informed consent to be included, and Institutional Review Board approval was secured for involvement of all human subjects. For the analysis that compares one eye with the other, data from both eyes were used. For the analysis that compares individual eyes between subjects, one eye was chosen at random.

**Figure 1:**
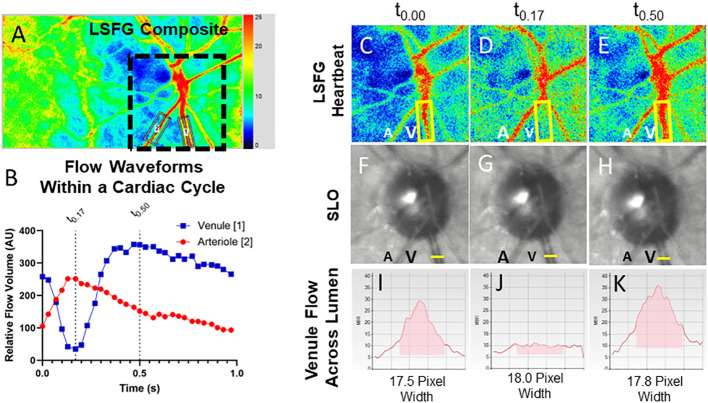
Relative volume flow (RFV) waveforms in a normal patient and in an eye with a CCF. Laser speckle flowgraphy simultaneously measures blood flow in retinal vessels enabling temporal comparison of a companion retinal arteriole **(A2)** and venule **(A1)**. The RFV waveform for an arteriole (red) and venule (blue) are shown for a representative eye affected by a CCF **(B)**. En-face blood flow maps **(C–E)** show that high-velocity flow (red pixels) disappears from the venule (V, yellow box) during systole while arterial flow A is maximal. Frames from a SLO video show that the venule does not collapse **(F–H)**, while cross-sectional flow measurement of the venule from LSFG **(I–K)** shows essentially no detectable intravascular flow when arterial flow is maximal [t0.17, **(J)**].

The LSFG 4-second video recordings were acquired in triplicate for each eye, from a LSFG-NAVI device (Softcare Ltd., Fukuoka, Japan). If the quality of the recordings was deemed to be unacceptable, the acquisition was repeated until an acceptable quality was achieved. Individual arterial and venous pulse waveforms were isolated by placing a “rubber band” region of interest along isolated companion pairs of major peripapillary retinal arterioles and venules (**Figure 1A**). The blood flow parameters of the arteriole–venule pairs were compared with a central retinal artery equivalent and a central retinal vein equivalent for validation (30). The RFV “heartbeat” waveform plots were extracted using Cobitos LSFG companion software (version 1.0.58.0; Softcare Ltd.) for waveform analysis.

The phase delay between the peak blood flow velocity in the retinal arterioles and venules (i.e., the A–V phase delay) was measured as the time elapsed between the peak of each waveform. It was expressed as a percent of the overall cardiac cycle duration. A higher percentage indicates greater asynchrony of the arteriole–venule velocity waveforms in each vascular bed. The A–V phase delay was calculated manually as:

(Frame # of the maximum venule RFV – Frame # of the maximum arteriole RFV)/Total number of frames in an average cardiac cycle * 100.

The greater positive values of a phase delay indicate a longer delay between the peak arteriolar flow velocity and the peak venular flow velocity. We compared A–V phase delays between affected and unaffected eyes in unilateral CCF cases, and because some cases were bilateral and had no internal control. We also compared all CCF eyes with a population of healthy control eyes. We then compared the phase delay of all CCF eyes to that of patients with papilledema, acute NAION, a central retinal vein occlusion (CRVO), and glaucoma. One-way ANOVA with a Tukey’s *post-hoc* test was used for statistical analysis. All statistical analysis was performed in GraphPad Prism v9.4.1 (GraphPad Software, San Diego, CA, USA). In the two CCF cases that we recorded scanning laser ophthalmoscope (SLO) video on a SPECTRALIS^®^ optical coherence tomography (OCT) device (Heidelberg Engineering, Franklin, MA, USA) to determine whether A–V phase delay is visible using technology widely available in ophthalmology offices. Pixel intensity changes over time (caused by vascular pulsation) were plotted using ImageJ (National Institutes of Health, Washington, DC, USA) from a segment of an arteriole and a companion venule to compare the timing of the pulsatile changes in reflectance at presentation and after treatment.

The waveform amplitudes of the companion arteriole and venule blood flow velocity were defined as the maximum RFV – minimum RFV for each isolated vessel within the cardiac cycles. The amplitudes were compared between the arterioles and venules in normal controls, CCF eyes, and unaffected fellow eyes using a Student’s *t*-test for analysis. To account for interindividual variation in the magnitude of RFV amplitudes, we normalized the venule amplitude by dividing it by the arteriole amplitude. This index (V: A amplitude ratio) was compared between normal subjects and the affected and unaffected eyes of unilateral CCF cases, using a Student’s *t*-test. We then compared the amplitude ratios between all CCF eyes, normal controls, and all patient groups using one-way ANOVA with a Tukey’s *post-hoc* test for statistical analysis.

## Results

### CCF cases, and baseline characteristics of control subjects and patients

**Figure 2:**
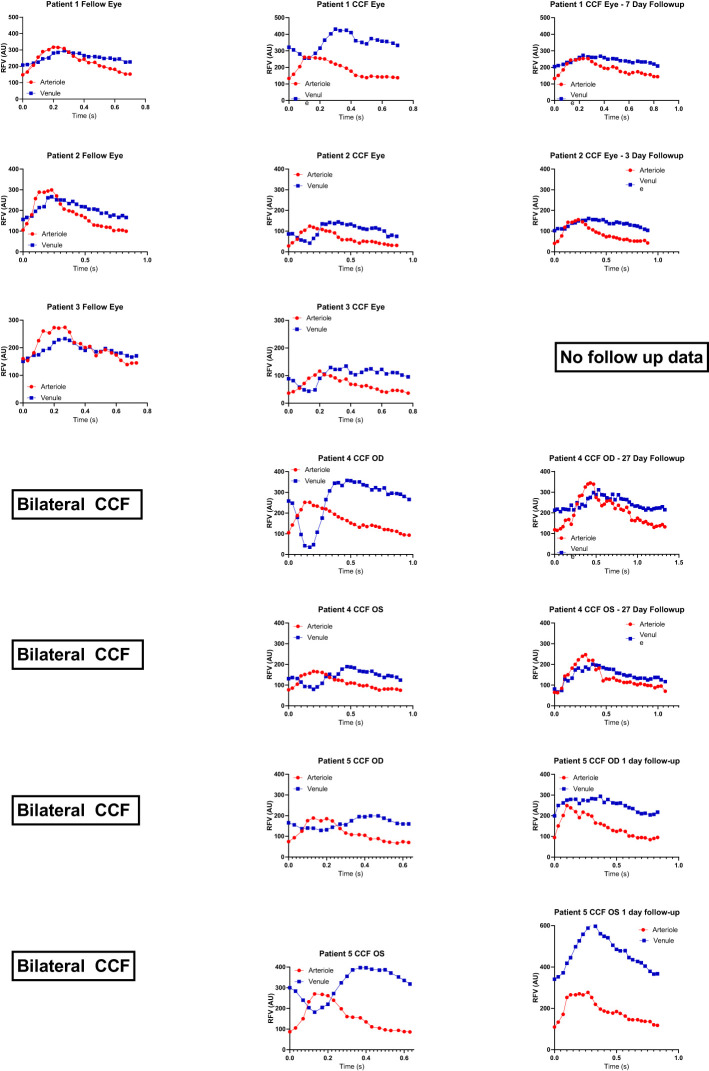
Relative volume flow (RFV) waveforms in CCF cases. RFV waveforms for arteriole and venule pairs in each CCF case are shown at presentation in the middle column. For unilateral cases, the unaffected fellow eye is shown in the first column to the left. For cases with follow-up recordings after thrombosis of the fistula, post-thrombosis scans are shown in the last column on the right. There is some degree of reduced venule blood flow velocity (blue) during peak arterial flow (red, corresponding to systole) in all cases of CCF. This was not observed in unaffected fellow eyes, nor after thrombosis of the fistula.

We enrolled five patients with CCFs from a single university neuro-ophthalmology practice (the RFV waveforms for each case are shown in **Figure 2**). Case 1 presented with classic features of a unilateral anterior-draining fistula with proptosis, painful ophthalmoplegia, corkscrew conjunctival vessels, mildly elevated IOP with pulsatility of the mires, and mildly tortuous retinal vessels. Case 2 presented with classic signs of a unilateral anterior-draining CCF with painful proptosis, ophthalmoplegia, dilated arterialized corkscrew vessels, elevated IOP with pulsatility of the mires on applanation, and venous stasis retinopathy consistent with impending CRVO. Case 3 presented with classic signs of a unilateral anterior-draining CCF with painful proptosis, ophthalmoplegia, dilated arterialized corkscrew vessels, elevated IOP with pulsatility of the mires on applanation, an orbital bruit, and dilated and tortuous retinal vessels. Case 4 presented initially with a unilateral abduction deficit from a posterior-draining fistula, followed by development of anterior drainage in both eyes causing proptosis, chemosis, and an elevated IOP, as well as progressive visual field loss in the more severely affected eye. Blood flow imaging was performed only after development of anterior signs. Case 5 presented with left greater than right exophthalmos, pulsatile ocular hypertension, pain, pulsatile tinnitus, diplopia, and a mild left optic neuropathy.

Four cases (1, 3, 4, and 5) had the presence of a CCF confirmed by digital subtraction angiography. Case 3 proceeded to spontaneously resolve after diagnostic angiography. Case 4 was treated successfully with transvenous coil embolization at the time of her diagnostic angiogram. In the other two cases, embolization at the time of diagnostic angiography was attempted, but was unsuccessful due to an anomalous venous anatomy, and so embolization was performed with a transorbital approach, with successful obliteration of the fistula. One patient had mild persistent arcuate visual field loss and diplopia (case 4). The other three (cases 1, 3, and 5) had complete resolution of their ocular symptoms. Case 2 did not have any follow-up following a spontaneous thrombosis and so her final outcome and post-thrombosis blood flow parameters are unknown.

**Figure 3:**
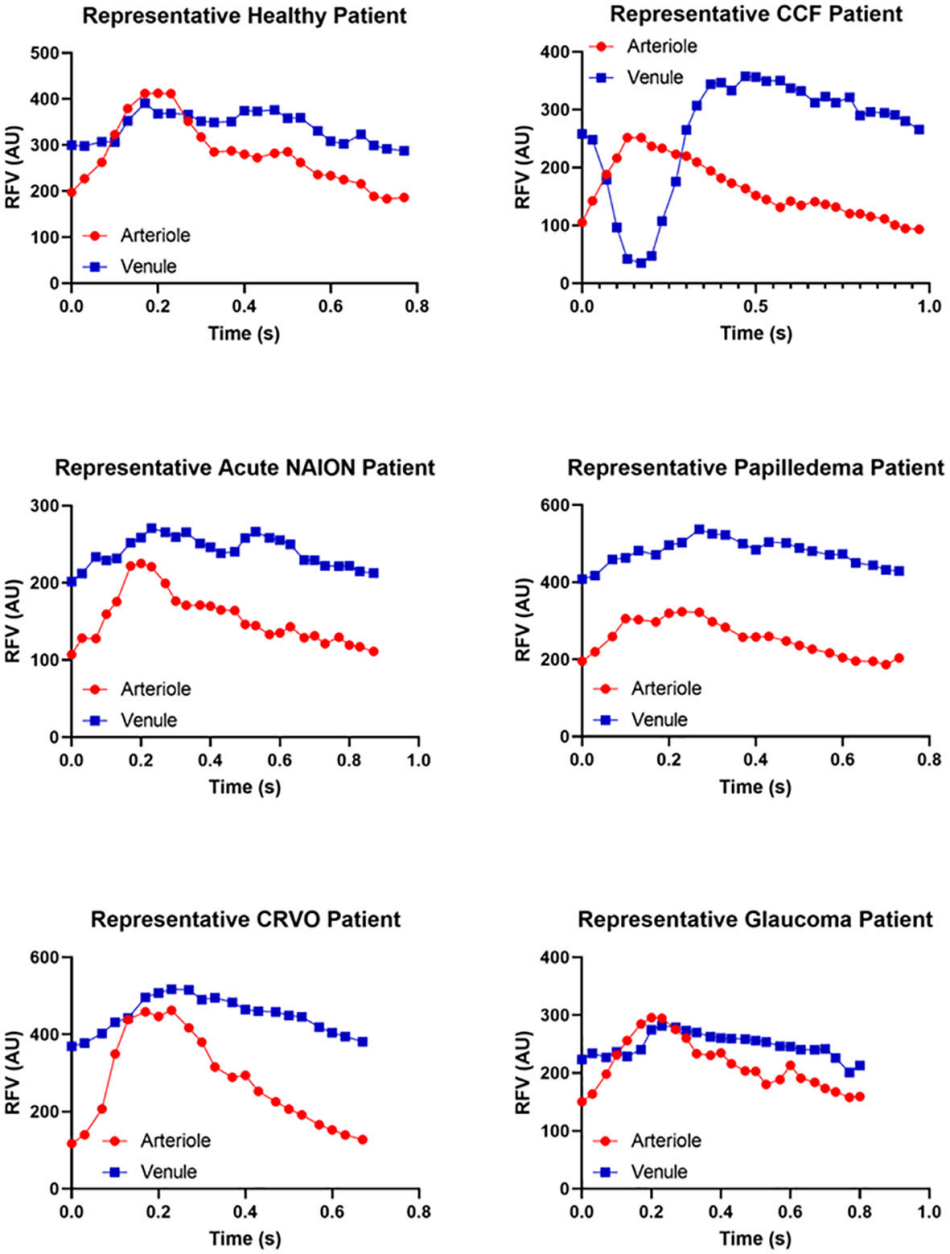
Representative relative volume flow (RFV) waveforms for each subject group. RFV waveforms show blood flow velocity changes over time within a single cardiac cycle. Representative waveforms measured within paired arterioles (red) and venules (blue) are shown for each patient group for reference and qualitative comparison. Note that the *y*-axis RFV scales are not matched between groups in order to accentuate the waveform shape. The A–V delay and increased venous pulsatility are not present in conditions without an A–V fistula, such as normal eyes, acute NAION eyes, papilledema, venous stasis retinopathy (CRVO), or glaucoma.

The patients with CCFs (*n* = 5) had a mean age of 69.5 years (range 51–92 years) and were all (100%) female. The normal subjects (*n* = 32) had a mean age of 47.3 years (range 18–74 years) and 78.1% were female. The control patients with CRVO (*n* = 3) were all male and had a mean age of 60.7 years (range 48–72 years). Patients with glaucoma (*n* = 4) were 50% female and had a mean age of 64.34 years (range 60–68 years). Eleven per cent of the patients with NAION (*n* = 23) were female and had a mean age of 63.5 years (range 40–95 years). Eighty percent of the patients with papilledema (*n* = 10) were female, with a mean age of 35.4 years (range 24–60 years) (**Table 1**). Representative waveforms of each patient group are shown in **Figure 3**.

**Table 1:** Summary of involved cases and patient demographics.

Characteristic	Primary diagnosis					
	Healthy patients (*n* = 32)	CCF (*n* = 5)	CRVO (*n* = 3)	Glaucoma (*n* = 4)	Acute NAION (*n* = 14)	Papilledema (*n* = 10)
Mean age, years (range)	47.3 (18–74)	70 (51–92)	60.7 (48–72)	64.3 (60–68)	59.4 (43–72)	35.4 (24–60)
Age (years), *n* (%)						
≥ 18 to < 60	26 (81.3%)	2 (40%)	1 (33.3%)	0 (0%)	7 (50%)	10 (100%)
≥ 60	6 (18.7%)	3 (60%)	2 (66.7%)	4 (100%)	7 (50%)	0 (0%)
Sex, *n* (%)						
Female	25 (78.1%)	5 (100%)	0 (0%)	2 (50%)	9 (64.3%)	8 (80%)
Male	7 (21.9%)	0 (0%)	3 (100%)	2 (50%)	5 (35.7%)	2 (20%)
Mean IOP, mmHg (range)	14.9 (9–21)	24.6 (20–28)	13.7 (11–16)	23.3 (11–44)	14.9 (9–21)	16.0 (11–22)
Mean blood pressure, mmHg (range)						
Systolic	127.8 (94–150)	143 (108–177)	147.7 (130–162)	131.0 (123–137)	142.5 (124–164)	130.3 (109–162)
Diastolic	79.0 (47–103)	85 (65–102)	94.7 (82–103)	81.3 (69–91)	77.1 (54–100)	83.2 (69–118)

### Blood flow analysis

**Figure 4:**
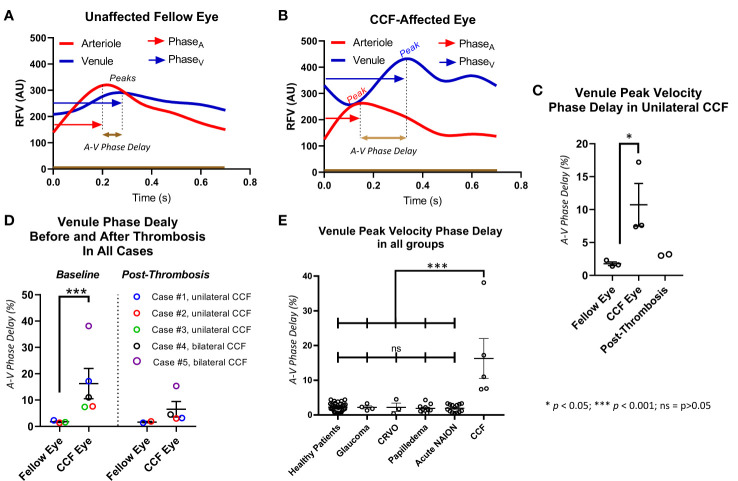
Delay in peak of venule relative flow volume. The venule peak flow delay is illustrated with a representative waveform from a healthy subject **(A)** and a patient with a carotid–cavernous fistula (CCF) **(B)**. The delay between arterial peak flow and venule peak flow is measured by measuring the time elapsed between the peaks and dividing that by the total time of the cardiac cycle. In cases with unilateral CCF **(C)**, and in all CCF cases **(D)**, the eye affected by the CCF had a significantly increased delay of retinal venous peak flow compared with arterial peak flow but not in the unaffected fellow eye, and this asynchrony improved after treatment. All eyes affected by CCF had a significantly greater venule peak flow delay than healthy controls, and patients with glaucoma, CRVO, papilledema, and acute NAION **(E)**.

The primary outcome, that is, the delay in peak blood flow velocity in the venule compared with the arteriole (A–V phase delay) was increased in the eyes affected by unilateral CCF (10.7% ± 0.2%; *n* = 3) compared with the unaffected fellow eyes (1.8% ± 0.2%; *n* = 3) (*p* < 0.05) (**Figure 4**). There was also a significant increase found in the A–V phase delay measured in all CCF eyes (10.8% ± 2.0%; *n* = 7) compared with the eyes of healthy control subjects (2.7% ± 0.3%; *n* = 32) (*p* < 0.001). After treatment, there was a substantial reduction in the A–V phase delay in the affected eyes, and eyes with thrombosed CCF (3.6% ± 0.4%) had no significant difference in the A–V phase delay compared with the fellow eyes (1.7% ± 0.2%) (*p* = 0.06),. Age had no effect on the A–V phase delay in the healthy control subjects (**Supplementary Table 1**), so we used the entire population of normal subjects as a control group for comparison with all CCF-affected eyes instead of a limited age-matched control group.

In each eye affected by CCF, in addition to a delay in the venule peak velocity, the venous RFV waveform showed that the flow in the venules was significantly reduced during systole, while the flow in the arterioles increased to its maximum during systole (**Figure 2**—RFV waveforms). The venular RFV appears to decrease during most of systole and then increases rapidly when the arterial flow slows down during diastole (**Figures 1F–H**).

The negative venous waveform deflection by LSFG disappeared in all cases after venous thrombosis and fistula closure (**Figure 2**) and was not seen in other conditions such as acute NAION, glaucoma, papilledema, and retinal vein occlusion (**Figure 3**).

**Figure 5:**
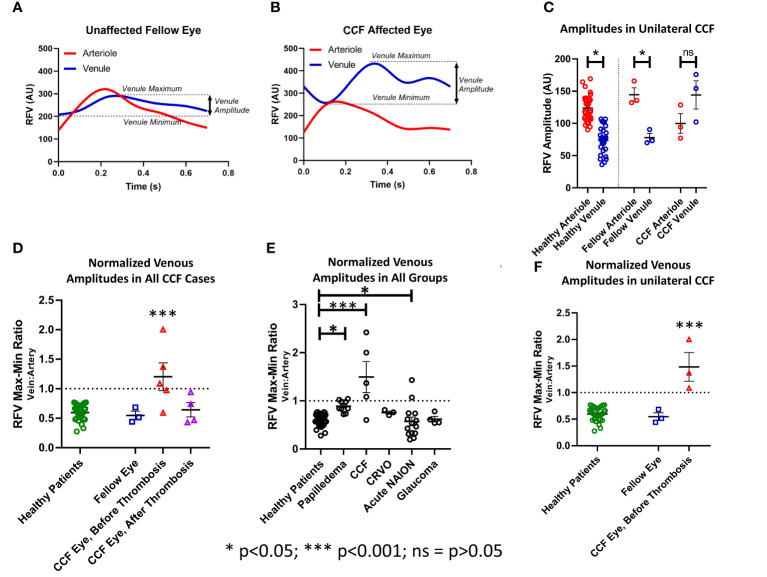
Pulsatility as measured by the relative volume flow (RFV) amplitude in venules and arterioles. The amplitude was defined as the difference between the maxima and minima of the RFV waveform for each arteriole and venule in each subject **(A, B)**. **(C)** The RFV values in arterioles were higher than in venules in healthy subjects and fellow eyes of the patients with unilateral CCF, but not for the CCF eyes themselves. **(F)** The ratio of vein-to-artery amplitude was significantly greater in the CCF eyes, and this effect improved after treatment **(D)**. Papilledema, acute NAION, and CCF patients all had greater vein-to-artery RFV amplitude ratios than the healthy controls **(E)**.

In normal subjects and unaffected fellow eyes, the RFV waveform analysis showed a blunted pulsatility, with less amplitude of change in the flow in the venules than in the companion arterioles, and represented a more constant flow in the venules (unaffected fellow eye venule amplitude 77.97 ± 5.21, arteriole = 144.73 ± 8.68, *p* < 0.01; normal control eye venule amplitude 73.83 ± 3.44, arteriole 123.71 ± 3.55, *p* < 0.001). In the eyes affected by CCF, this relationship changed significantly (**Figures 4**, **5**), as the artery and vein showed similar, but asynchronous flow velocity pulsatile waveform amplitudes (venule 144.13 ± 17.86; artery 99.93 ± 12.34; *p* = 0.17). Normalizing the venule amplitude to the arteriole amplitude revealed a significant increase in the venule-to-arteriole ratio in CCF eyes compared with the normal control eyes. (V: A amplitude CCF 1.48 ± 0.38, healthy 0.60 ± 0.02; *p* < 0.001) After treatment, the pulsatility of the venular flow diminishes and there is no difference from normal patients (CCF post-treatment A: V RFV ratio 0.66 ± 1.9; *p* < 0.17) (**Figure 5**). Among the control patients with other conditions than CCF, papilledema patients also showed an increased V: A amplitude ratio compared with healthy controls, albeit to a lesser extent than in CCF (papilledema V: A RFV amplitude ratio 0.89 ± 0.03, healthy 0.60 ± 0.2; *p* = 0.011 by one-way ANOVA).

### Scanning laser ophthalmoscopy analysis

**Figure 6:**
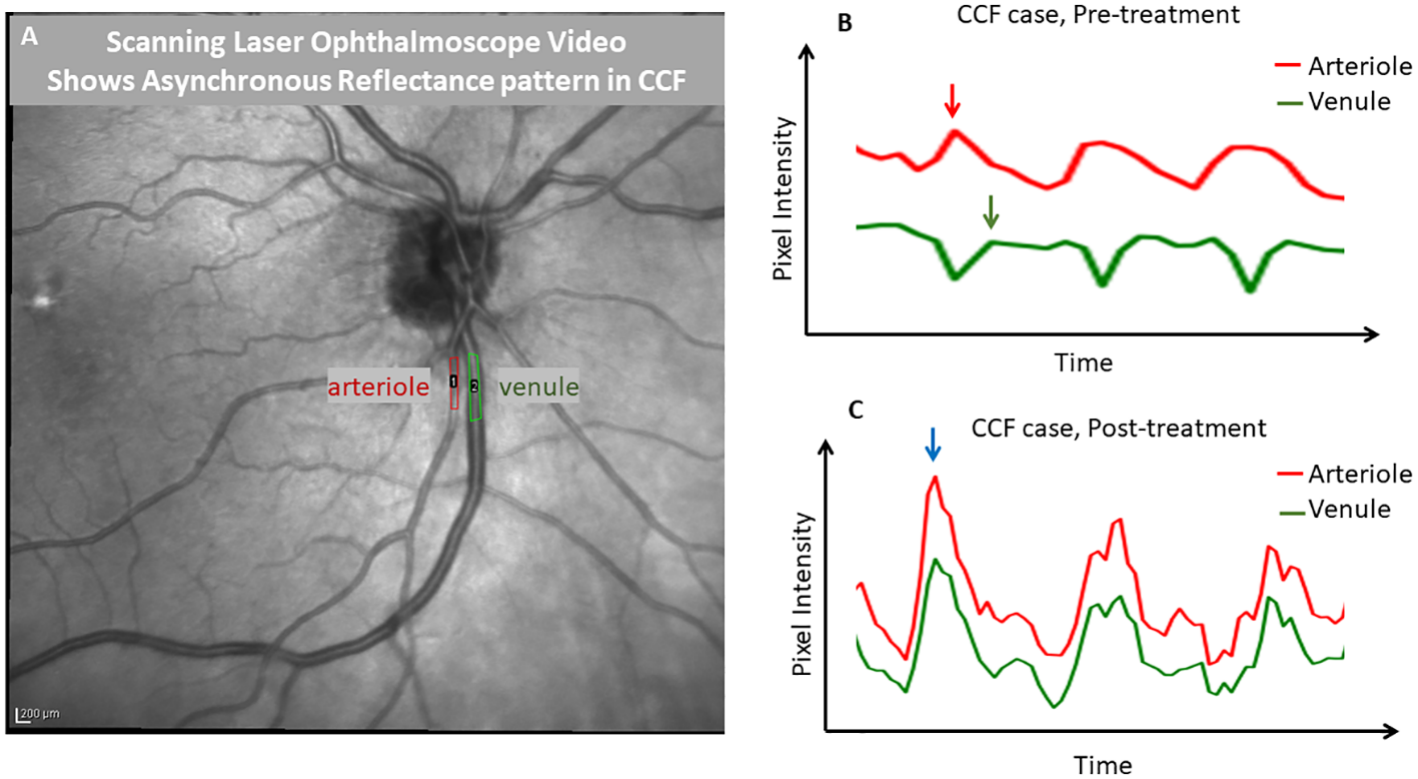
Demonstration of phase delay that is visible with scanning laser ophthalmoscopy. A frame from an infrared scanning laser ophthalmoscope video **(A)** captured with a Heidelberg SPECTRALIS OCT is shown, with regions of interest drawn over a major retinal arteriole and venule. ImageJ was used to extract a pixel intensity waveform from each region of interest to show the relative timing of the changes in the light reflex over the vessels. Before treatment of the carotid–cavernous fistula **(B)**, the venule tracing in green showed a reduction in pixel intensity, while the arterial pixel intensity was maximal (during systole). However, after treatment **(C)**, the reflexes in both vessels were synchronized and pixel intensities changed in parallel. This demonstrates that SLO can be used to identify the finding of an arteriovenous pulse delay. This effect was quite visible with the video alone and in an unaided observation; the tracing is shown for further demonstration.

Images from an en-face SLO video in a case of CCF showed no significant change in the vessel diameter throughout the cardiac cycle, even in the most extreme cases when the flow in the veins reached a standstill; there was no evidence of venous collapse. However, when the pixel intensity over a retinal arteriole and companion venule was analyzed and then graphed as a function of time, the pixels within the arteriole became brighter during systole, while the pixels over the companion venule changed in the opposite direction, becoming darker (**Figure 6**). After thrombosis, the pixel intensity increased in both the arteriole and venule during systole. This pattern of opposing pixel intensity in the arteriole and venule has not been seen in eyes without CCF.

## Discussion

In normal eyes, venous outflow velocities appear temporally coupled to arterial inflow velocities, and the velocity of flow varies much less in the venous system than it does in the arterial system. In normal eyes, an increase in arterial inflow during systole results in a synchronous increase in the venous outflow, with no sign of resistance to venous drainage in the retinal venules. Ocular plethysmography in eyes with a CCF has previously demonstrated that there is a wider ocular systolic pulse amplitude of the eyeball and a lower ocular perfusion pressure than control eyes. This was suggested to indicate significant shunt physiology of large-volume changes in the ocular vasculature during the cardiac cycle, but reduced blood flow due to a reduction in AV pressure difference (20). Proximal AV shunting in the cavernous sinus may result in a phasic increase in the venous pressure in the valveless orbital and retinal veins that is maximal during systole. This could reduce the AV pressure difference, thus reducing systolic blood flow in both the arterioles and venules. In our CCF cases, we did find reduced venous blood flow velocity during systole to a variable degree, but systolic arterial pressure still appears to drive blood into the eye despite poor outflow. The blood is probably filling venous capacitance vessels until the outflow resistance abates during diastole, something that is not likely to occur in other conditions with a more constant venous congestion. After systole, the venous blood may maximally dissipate, reaching a velocity peak during diastole, also at a time when IOP is at its lowest. This may be one explanation for the combination of reduced arterial and venous flow during systole and the delayed peak blood flow in the venules during diastole. The result is that proximal AV shunting in the cavernous sinus causes changes in venous capacitance and other hemodynamic changes that result in a characteristic temporal decoupling of peak arterial and venous flow within the eye that was observed in CCF.

In the eyes affected by a CCF, we found that there was a measurable delay in the peak flow velocity in the retinal venules with respect to the retinal arterial peak flow velocity (A–V phase delay), which reverses after treatment or on spontaneous resolution of the fistula (**Figure 4**). This was not present in the unaffected eyes of unilateral cases, nor was it found in the normal subjects or eyes with papilledema, NAION, CRVO, or glaucoma. The delay was also visible as asynchronous oscillations in the reflectance, which was observed on careful inspection of the SLO video in the cases where that was obtained (*n* = 2). There was also an obvious amplitude change in the venular RFV pulse waveform, especially when normalized to arterial pulse amplitude, owing in part to a mid-systolic decrease in venular flow that was observable on qualitative inspection of the waveforms. This invisible but measurable perturbation of retinovascular flow also reversed after treatment or on spontaneous resolution of the fistula (**Figure 5**).

We considered whether or not retinal vein compression could account for the mid-systolic decrease in venular flow and found that an en-face SLO video showed no venous collapse (**Figure 1**). There were no substantial changes in venule diameter throughout the cardiac cycle, suggesting that the RVF decrease does represent slowed flow rather than extrinsic venule compression and collapse. The combination of increased venular phase delay and pulse amplitude increase is not seen in other conditions that affect venous drainage from the retina in a more static manner, such as with optic disc edema or retinal vein occlusion The asynchrony in retinal arterial and venous blood flow appears to be a sensitive and specific marker of CCF, but its diagnostic usefulness needs to be more fully explored in more cases. Our preliminary data are promising, especially given the consistent restoration of AV flow synchrony with treatment and venous thrombosis. Our study of intravascular waveform characteristics was able to identify flow signatures of an underlying fistula that can be readily measured at the point of care by LSFG, or with a more widely available SLO/OCT instrument by recording a video of the retinal vessels during the cardiac cycle.

Our study is limited by sample size due to the relatively low incidence of CCF, and the number of subjects with different pathologies in our retrospective LSFG dataset. However, even with the number of subjects studied, there were still statistically significant differences between affected and fellow eyes and between groups. One eye in our cohort (case 2) had an impending CRVO complicating the CCF, which could confound the analysis of the effect of phase delay if a CRVO increased the phase delay. When we looked at CRVO cases that were not due to a CCF, we saw no increased A–V phase delay, so we felt that it was reasonable to include that case in the primary analysis. This also raises the possibility that an increased A–V phase delay could possibly identify the rare case of CRVO that is actually caused by a CCF.

We conclude that a decrease in venule velocity during systole with an increased but delayed venous pulsation, asynchronous with arterial pulsation, appears to be diagnostic for a CCF. Given that the gold standard for diagnosis of CCF is digital subtraction angiography, which carries around a 1% risk of cerebral vascular accident, it would be worthwhile to find inexpensive, non-invasive markers that could be used to diagnose, follow, and risk stratify these challenging patients.

## Data availability statement

The raw data supporting the conclusions of this article will be made available by the authors, without undue reservation.

## Ethics statement

The studies involving humans were approved by Institutional Review Board at the University of Iowa Hospitals and Clinics. The studies were conducted in accordance with the local legislation and institutional requirements. The participants provided their written informed consent to participate in this study.

## Author contributions

EL: Formal Analysis, Investigation, Methodology, Writing – original draft. TT: Formal Analysis, Investigation, Visualization, Writing – review & editing. N-U-SA: Formal Analysis, Investigation, Visualization, Writing – review & editing. J-KW: Formal Analysis, Software, Visualization, Writing – review & editing. RK: Conceptualization, Resources, Supervision, Writing – review & editing.
